# Effect of Pre-Fermentative Maceration and Fining Agents on Protein Stability, Macromolecular, and Phenolic Composition of Albariño White Wines: Comparative Efficiency of Chitosan, k-Carrageenan and Bentonite as Heat Stabilisers

**DOI:** 10.3390/foods10030608

**Published:** 2021-03-12

**Authors:** Inma Arenas, Miguel Ribeiro, Luís Filipe-Ribeiro, Rafael Vilamarim, Elisa Costa, João Siopa, Fernanda Cosme, Fernando M. Nunes

**Affiliations:** 1 Chemistry Research Centre-Vila Real (CQ-VR), Food and Wine Chemistry Lab., University of Trás-os-Montes and Alto Douro, 5000-801 Vila Real, Portugal; inmarenas@hotmail.com (I.A.); jmribeiro@utad.pt (M.R.); fmota@utad.pt (L.F.-R.); rafa_vilamarim_93@hotmail.com (R.V.); ecmatos@gmail.com (E.C.); joaosiopa2109@hotmail.com (J.S.); fcosme@utad.pt (F.C.); 2 Biology and Environment Department, School of Life Sciences and Environment, University of Trás-os-Montes and Alto Douro, 5000-801 Vila Real, Portugal; 3 Chemistry Department, School of Life Sciences and Environment, University of Trás-os-Montes and Alto Douro, 5000-801 Vila Real, Portugal

**Keywords:** Albariño white wine, k-carrageenan, fungal chitosan, sodium bentonite, calcium bentonite, protein stability, protein profile, polysaccharides, chromatic characteristics, phenolic compounds

## Abstract

In this work, the effect of pre-fermentative skin maceration (PFSM) on the chemical composition of the macromolecular fraction, polysaccharides and proteins, phenolic compounds, chromatic characteristics, and protein stability of Albariño monovarietal white wines was studied. PFSM increased the extraction of phenolic compounds and polysaccharides and reduced the extraction of pathogenesis-related proteins (PRPs). PFSM wine showed significantly higher protein instability. Sodium and calcium bentonites were used for protein stabilisation of wines obtained with PFSM (+PFSM) and without PFSM (−PFSM), and their efficiencies compared to fungal chitosan (FCH) and k-carrageenan. k-Carrageenan reduced the content of PRPs and the protein instability in both wines, and it was more efficient than sodium and calcium bentonites. FCH was unable to heat stabilise both wines, and PRPs levels remained unaltered. On the other hand, FCH decreased the levels of wine polysaccharides by 60%. Sodium and calcium bentonite also decreased the levels of wine polysaccharides although to a lower extent (16% to 59%). k-Carrageenan did not affect the wine polysaccharide levels. Overall, k-carrageenan is suitable for white wine protein stabilisation, having a more desirable impact on the wine macromolecular fraction than the other fining agents, reducing the levels of the wine PRPs without impacting polysaccharide composition.

## 1. Introduction

In winemaking, pre-fermentative skin maceration (PFSM) is a process applied to white wine production where the skins of crushed and destemmed white grapes are macerated in their juice at controlled conditions (time and temperature) before pressing. This process aims to obtain the maximum intensity of varietal aroma and to improve white wine quality [1,2]. Nevertheless, skin maceration also increases the levels of phenolic compounds extracted increasing wine bitterness, astringency, and browning potential [3,4,5]. Also, the higher levels of potassium ions extracted enhance potassium bitartrate precipitation, reducing tartaric acid concentration, resulting in lower titratable acidity and higher pH, and consequently perceived sourness is diminished [6,7,8]. Ramey et al. [3] using different maceration temperatures (9 °C and 15 °C) showed that the wine pH increased from 3.33 to 3.49, respectively, and this would affect the protein stability, also the protein concentration increased 50% (21.9 to 31.6 mg/L). Likewise, Tian et al. [5] showed that the amount of protein increased in juice obtained with PFSM, particularly the pathogenesis-related proteins (PRPs).

PRPs are the main determinants of white wine protein instability, being grape-derived proteins with high molecular weight and low isoelectric point, and include the chitinases and thaumatin-like proteins [9,10,11]. These proteins can be responsible for a wine’s colloidal instability, producing a suspended and undesirable haze, and forming amorphous sediment or flocculate, before or after bottling [9,12,13,14,15]. This instability can cause serious economic losses to the wine producers. Wine protein haze formation can occur under high temperatures, throughout storage, or wine transportation due to the protein self-aggregation phenomena, resulting in light-dispersing particles [16]. Protein instability can also occur through the blending of protein stable wines. This phenomenon needs to be prevented by removing them from the wine, usually by fining, before wine bottling [17,18]. Bentonite fining is the most effective and used process to avoid protein instability in white wine [19,20,21]. However, bentonite fining can affect wine quality, for example, by removal of colour, aroma, and flavour compounds [14,22,23,24,25] affecting the wine sensory characteristic [26]. Also, the application of high doses of bentonite produces bentonite lees that can comprise 5% to 20% of the wine volume associated with the poor settling characteristics [27] and presents additional waste disposal challenges [14,28]. Therefore, alternative techniques to bentonite fining for this goal have been studied, such as chitin and chitosan [29,30], carrageenan [15,31], and the use of mannoproteins [32,33]. The use of chitosan in winemaking has been authorised by the European Union (EU) for heavy metals and contaminant removal, prevention of cloudiness, and reduction of undesirable *Brettanomyces* spp. population [34]. For wine applications, only chitin (Oeno 367-2009 Chitin-Glucan [35]) and chitosan (Oeno 368-2009 Chitosan [35]) obtained from the cell walls of *Aspergillus niger* or *Agaricus bisporus* are authorised. Fungal chitin shows some special features, concerning the chemical structure and biosynthesis [36] when compared to crustacean chitins. However, a major difference results from the fact that fungal chitin is associated with other polysaccharides which do not occur in the exoskeleton of arthropods [36,37]. Furthermore, chitins and chitosans can present a diversity of structural features like their deacetylation degree and molecular weight that can affect their properties like charge density and solubility [36,37]. Chitin can remove specific wine proteins, namely the grape class IV chitinases [38]. The application of 1 g/L of chitin reduced the wine haze induced by the heat test by 50%, while the application of 20 g/L of chitin reduced the haze by 80%. This haze reduction observed was directly linked to the elimination of the class IV grape chitinases. Colangelo et al. [30] also observed that wines treated with 1 g/L of fungal chitosan–glucan improved heat stability at 55–62 °C and this was also due to the specific elimination of chitinases. On the other hand, carrageenan due to their negative charge at low pH can electrostatically flocculate and sediment positively charged proteins responsible for wine protein instability [15,31,39,40]. Cabello-Pasini et al. [39] showed that carrageenan removed the same protein fractions adsorbed by bentonite, establishing that carrageenan might have a higher wine stabilisation efficiency without changing the wine tannin concentration when compared to bentonite fining. Marangon et al. [15,40], also observed that carrageenan has no adverse sensory impacts compared to wine fined with bentonite. In addition, the wine treated with carrageenan did not increase lees volume comparative to bentonite fining. Likewise, Ratnayake et al. [31] used commercially carrageenan’s to understand the protein stabilisation efficiency and the impact on wine sensory characteristics. From the different commercial carrageenan’s tested two of them were able to heat (80 °C/2 h, 20 °C/3 h) stabilise the white wine without negative impacts on the wine’s sensory characteristics. However, if carrageenan is applied to the wine, there is a risk of obtaining wines that are unstable by performing the protein stability (heat test) due to carrageenan remaining in wine, contributing to haze formation in the heat test and also the wine presenting a lower filterability mainly when carrageenan is applied before or during fermentation [15,40]. According to Ratnayake et al. [31], the wine filterability differs with the carrageenan structure and time of application. Therefore, the application of carrageenan’s for white wine protein stabilisation has shown to be effective when applied either during wine processing or to the finished wine [15,39,40]. Although the impact of these fining agents on protein concentration has been studied, their impact on wine polysaccharides, the other macromolecular component of wines, is largely unknown. Wine polysaccharides have their origin in grape cell walls and comprise polysaccharides rich in arabinose and galactose (arabinans, type I arabinogalactans, and type II arabinogalactan proteins) and type I and type II rhamnogalacturonans [41,42]. These polysaccharides are released in white winemaking when grape skins and grape cell solids are in contact with the juice before fermentation [41,42]. Another wine polysaccharides are derived from the yeast cell walls during fermentation and ageing on lees, such as mannoproteins and glucans [41]. The amount and type (chemical composition, molecular structure, and origin), of wine polysaccharides, influence wine properties and sensory characteristics [43,44]. Wine polysaccharides are important to stabilise other molecules, avoiding or delaying aggregation and flocculation and consequently haze formation [45,46]. According to Gawel et al. [42], the wine polysaccharide structures’ diversity and complexity give them the ability to form intermolecular associations with other wine compounds responsible for wine mouthfeel either through hydrogen bonding or hydrophobic interactions. The white winemaking operations related to juice extraction such as pre-fermentation skin maceration, skin, and whole-bunch pressing, yielded differences in the polysaccharide’s concentration around 15% [47]. However, the effect of polysaccharides on white wine mouthfeel and the taste was slight compared with that of the wine matrix components of alcohol and pH [42].

Thus, this work aimed to study the effect of the skin contact on Albariño monovarietal white wines obtained with and without pre-fermentative skin maceration on the wine macromolecular fractions, proteins and polysaccharides, phenolic compounds, chromatic characteristics, and protein stability. Also, the efficiency of fungal chitosan and k-carrageenan on wine protein stability as alternative fining agents to sodium and calcium bentonites were studied, as well the impact on the wine polysaccharides.

## 2. Materials and Methods

### 2.1. Winemaking Technology and Wine Composition

The Albariño monovarietal white grapes, used in this study for the production of the white wines remained in the harvest boxes for 10 h in a cold chamber at 5 °C. The grapes were then destemmed/crushed and potassium metabisulfite (10 g/hL, Agrovin, Ciudad Real, Spain), enzymes (pectin lyase and polygalacturonase, 0.4 mL/hL, Enozym Lux, Agrovin, Ciudad Real, Spain), and hydrolysable gallic tannin (3 g/hL, Galitan, Agrovin, Spain) were added. To produce the white wine without pre-fermentative skin maceration (−PFSM), the crushed grapes were pressed in an inert atmosphere of CO_2_ with the addition of enzymes (pectolytic enzymes, pectin lyase, polygalacturonase, pectinesterase, and cellulases, 2 g/hL, Enozym Éclair-AEB). Then the clarified grape juice inoculated with active dry yeasts (*Saccharomyces* spp., 20 g/hL, Viniferm Elegancia, Ciudad Real, Spain), and the alcoholic fermentation was performed at a controlled temperature (14−15 °C). At 1080 specific gravity the grape must was supplemented with ammonium phosphate, inactivated yeasts, and thiamine at 5 g/hL (Actimax Plus, Agrovin, Ciudad Real, Spain) and at 1054 specific gravity supplemented with organic nitrogen, and vitamins at 10 g/hL (Actimax Vit, Agrovin, Ciudad Real, Spain). The alcoholic fermentation finished when residual sugars were <2 g/L. For the Albariño monovarietal white wine with pre-fermentative skin maceration (+PFSM) after destemming/crushing and before pressing the grape juice remained in contact with the grape skin and seeds, during 8 h at a temperature of 5 °C, all the other operations were unchanged. The final wine oenological parameters shown in Table 1 were analysed using a Fourier transform infrared spectroscopy (FTIR) Bacchus Micro (Microderm, Paris, France). The wine was stored in stainless steel tanks of 7000 litters protected with sulphur dioxide at 50 mg/L (free sulphur dioxide).

### 2.2. Fining Experiments

Albariño monovarietal white wine obtained without pre-fermentative skin maceration (−PFSM) and with pre-fermentative skin maceration (+PFSM) were fined with fungal chitosan (No Brett Inside, Lallemand, Midi-Pyrénées, France; 100 g/hL), within the limits allowed by the International Organisation of Vine and Wine (OIV) to avoid instabilities [34], k-carrageenan (CEAMSA, Pontevedra, Spain; 100 g/hL), sodium bentonite (SAIstab^®^BENTO MDP, SAI Enology, Paredes, Portugal; 120 g/hL), and calcium bentonite (SAIstab^®^BENTO CLS, SAI Enology, Paredes, Portugal; 120 g/hL) using the maximum dosage recommended by the manufacturer, since there are no official limits for their application in wines. Before use, sodium and calcium bentonites were hydrated in water for 8 h at a ratio of 1:20. Fungal chitosan was dispersed in water at 3.125% before use. k-Carrageenan was added directly to the wine. The fining experiments were conducted in 250 mL graduated cylinders by mixing the wine with the fining agent and allowing the mixture to remain in contact with the wines for 7 days at 20 °C, simulating the standard oenological practices. Before fining the level of free sulphur dioxide was adjusted to 50 mg/L, if needed, and the graduate cylinders were closed with Parafilm^®^M (Merck, Darmstadt, Germany). Wine samples without pre-fermentative skin maceration (−PFSM) and with pre-fermentative skin maceration (+PFSM) without the addition of a fining agent were used as a control. The samples were centrifuged at 537.6 g for 15 min before analysis. All experiments were performed in duplicate.

### 2.3. Macromolecular Material Isolation

For the isolation of the wine macromolecular material that includes wine protein and polysaccharides, to 100 mL of controls and treated-samples was added urea to a final concentration of 6 M. This initial step was conducted to reduce non-covalent interactions between proteins and polyphenols. Then, this mixture was subjected to ultrafiltration (molecular weight cut-off 10 kDa) up to the volume of approximately 5 mL [48]. To remove urea from the solution, this volume was made up to 100 mL with ultrapure water at least 5 times. Finally, a volume of about 10 mL was recovered and freeze-dried yielding the wine macromolecular material.

### 2.4. Electrophoresis (Sodium Dodecyl Sulphate Polyacrylamide Gel Electrophoresis (SDS-PAGE)

Wine macromolecular material, containing wine proteins, was diluted in sample buffer containing 2% (*w*/*v*) sodium dodecyl sulphate (SDS), 40% (*v*/*v*) glycerol, 0.02% (*w*/*v*) bromophenol blue, 0.08 M Tris-HCl pH 8.0, heated at 70 °C during 10 min and separated in a resolving gel with 16% total monomer concentration (T) using a Hoefer SE 600 Ruby unit (Amersham Biosciences, Uppsala, Sweden) at 30 mA/gel. The gels were stained with Coomassie Blue R-250 for 24 h and then washed in distilled water overnight.

### 2.5. Protein Quantification

Wine total protein concentration was determined by the Bradford assay modified by Read and Northcote [49]. Briefly, dye-reagent was prepared using 0.01% (*w*/*v*) Serva Blue G (Heidelberg, Germany) in 1.6 M phosphoric acid/0.8 M ethanol. The assay was performed in 1-mL glass cuvette, which was washed with ethanol followed by distilled water between samples. For wine macromolecular material, containing wine proteins, dye-reagent (950 µL) was added to 50 µL of protein solution (0.5% *w*/*v* in ultrapure water). In the case of direct analysis of wines, dye-reagent (1000 µL) was added to 200 µL of white wines. After 4 min absorbance was measured at 595 nm. Samples were assayed in duplicate. Development of a standard curve for the semi-quantitative procedure was done using increasing bovine serum albumin (BSA) concentrations. Glass tubes were always chosen instead of plastic tubes.

### 2.6. Reversed Phase-High Performance Liquid Chromatography (RP-HPLC) Quantitative Protein Analysis

Wine macromolecular material, containing wine proteins, was solubilized in ultrapure water (0.5% *w*/*v*) and separated by reversed phase-high performance liquid chromatography (RP-HPLC). For RP-HPLC analysis, an RP-C8 column was used (25 cm, 4.5 mm internal diameter (i.d.), 5 l m, Macherey-Nagel, Germany) maintained at 35 °C during the separation process, and an injection volume of 100 µL was used. A gradient elution was performed; eluent A consisting of 0.1% (*v*/*v*) aqueous trifluoroacetic acid and eluent B consisting of acetonitrile and trifluoroacetic acid (99.9/0.1%, *v*/*v*), with following elution program: 0 min 20% B, 7 min 40% B, 15 min 50% B, 16 min 55% B, 30 min 66% B, 35 min 66% B, 36 min 20%B, 42 min 20% B; flow rate of 1 mL/min. Detection was made by ultraviolet (UV) absorbance at 210 nm. Development of a standard curve for the RP-HPLC quantitative procedure was conducted using increasing BSA concentrations. Protein identification was performed using the data reported in the literature [50,51].

### 2.7. Protein Heat-Stability Test

The efficacy of different fining agents (sodium bentonite, calcium bentonite, fungal chitosan, and carrageenan) in producing heat-stabilised wines were assessed in trials with an Albariño monovarietal white wine obtained without pre-fermentative skin maceration (−PFSM) and with pre-fermentative skin maceration (+PFSM). Samples were filtered at 0.45 μm (Ultipor N66, Pall Corporation, New York, NY, USA) and assessing the heat stability of each sample (80 °C for 30 min) according to Dubourdieu et al. [52]. If the difference (ΔNTU) in nephelometric turbidity unit (NTU), between the heated and unheated samples, was higher than 2 NTU units, means that the wine sample is unstable [52]. All analyses were performed in duplicate.

### 2.8. Filterability Index

The filterability index of the wines was measured according to Descout et al. [53]. Wine (approximately 700 mL) was added to a stainless-steel chamber and filtered through a disc filter [0.45 μm polyethersulfone membrane, Millipore Express] under pressure (2 bar) and collected in a measuring cylinder. The time in seconds taken to filter 200 mL and 400 mL of wine was recorded to calculate the filterability index (FI), where FI > 20 was indicative of filtration issues. Analyses were performed in triplicate.

FI = T400 − 2T200

### 2.9. Quantification of Non-Flavonoids, Flavonoids and Total Phenols

The phenolic content of the wines was quantified using the absorbance at 280 nm before and after precipitation of the flavonoid phenols, through reaction with formaldehyde, according to Kramling and Singleton [54]. The non-flavonoid phenolic compounds of the white wine were quantified according to Kramling and Singleton [54], and the total phenolic compounds, were also determined by a spectrophotometric method, using an ultraviolet–visible (UV–vis) spectrophotometer according to Ribéreau-Gayon et al. [55]. The flavonoid phenolic compounds were obtained by the difference between total phenolic compounds and non-flavonoid phenolic compounds [54]. Quantifications were performed using the calibration curve of gallic and the results were expressed as gallic acid equivalents/L. All analyses were performed in duplicate.

### 2.10. Chromatic Characteristics and Colour (A_420 nm_)

Absorption spectra of wine samples were scanned from 380 nm to 780 nm, using a 1 cm path length quartz cell, and the chromatic characteristics of wines L* (lightness), a* (redness), and b* (yellowness) coordinates were calculated using the CIELab colour space method according to OIV [56]. The colour difference between the wine sample after oxidation and the control wine was calculated using the following equation: ΔE*= [(ΔL*)^2^ + (Δa*)^2^ + (Δb*)^2^]^1/2^. Colour differences higher than 2 units can be distinguished by the human eye [57]. The white wine colour was determined by measuring absorbance at 420 nm (1 cm cell) as described in the OIV methods [56]. All analyses were performed in duplicate.

### 2.11. High-Performance Liquid Chromatography (HPLC) Analysis of Catechin and Phenolic Acids

The analysis of phenolic acids and catechin of white wines was performed by Reversed Phase (C-18 stationary phase, 250 mm × 4.6 mm, 5 μm particle size, ACE, Scotland) High Performance Liquid Chromatography (Ultimate 3000 Dionex, Thermo Fisher Scientific, Waltham, MA, USA) with photodiode array detection (200 to 650 nm, PDA-100, Ultimate 3000 Dionex, Thermo Fisher Scientific, Waltham, MA, USA). Before analysis the wine was concentrated 25 fold by vacuum evaporation (50 mL of wine:2 mL of methanol:water 1:1), and a 50 μL injection volume was used. During separation the column temperature was set at 35ºC and a 1 mL/min flow rate was used. The elution was performed using 5% aqueous formic acid (A) and methanol (B) and the following gradient was used: 5% B (0–5 min); 5–65% B (5–65 min); 65% to 5 % (65–67 min) [58]. Quantification was performed with calibration curves with pure commercial standards when available (caffeic acid, coumaric acid, ferulic acid, gallic acid, and catechin). The calibration curve of caffeic acid was used for the quantification of *trans*-caftaric acid, 2-*S*-glutathionylcaftaric acid (GRP), and caffeic acid ethyl ester. The calibration curve of *p*-coumaric acid was used for the quantification of coutaric acid and coumaric acid ethyl [59,60]. Analyses were performed in duplicate.

### 2.12. Polysaccharides Sugar Composition and Content

The sugar composition of wine polysaccharides was determined after acid hydrolysis with 1M H_2_SO_4_ at 100 °C during 2.5 h by anion-exchange chromatography with pulsed amperometric detection (ICS-3000, Dionex, with an electrochemical detector (ED) containing an Au working electrode, Ag/AgCl reference electrode, and Ti counter electrode) according to Fraga and Nunes [61].

Quantification was performed by the internal standard method using 2-deoxyglucose as internal standard and the calibration curve method (0.25–2.5 mg of sugar/0.5 mg of internal standard) with pure commercial standards of fucose, rhamnose, arabinose, galactose, glucose, mannose, xylose, galacturonic, and glucuronic acid standards. Separation was performed with a CarboPac PA-20 column (150 mm × 3 mm) with a CarboPac PA20 pre-column (Dionex, Sunnyvale, CA, USA) maintained at 35 °C during the run. The eluents were keep under nitrogen. Elution was performed using three solutions: eluent A – 1.25 mM NaOH solution containing 2 mM Ba(OH)_2_, eluent B – 400 mM sodium acetate containing 2 mM Ba(OH)_2_ and eluent C – 500 mM NaOH containing 2 mM Ba(OH)_2_, using the following elution program: 0–19 min, 100% A, 0%B, 0%C, 19–27 min 50%A, 50% B, 0% C, 27–37 min; increase to 10%A, 50%B, 40% C; 37–47 min 60%A, 0% B, 40% C and maintained until 57 min. The column was conditioned with 100% A, 0%B, 0% C for 15 min before injection. The injection volume was 5 μL, the flow rate was 0.3 mL/minThe ED cell waveform was +0.1 V from 0.00 to 0.40 s, then −2.0 V from 0.41 to 0.42 s, and a ramp −2.0 to +0.6 V from 0.42 to 0.43 s, followed by −0.1 V from 0.44 to 0.50 s (end of cycle). The integration region was from 0.2 to 0.4 s. All analyses were performed in duplicate.

### 2.13. Statistical Analysis

The results are presented as means ± standard deviation. Physicochemical data were statistically tested by the Student *t*-test when comparing two independent samples and by analysis of variance (ANOVA) when comparing more than two independent samples. Tukey’s honestly significant difference (HSD, 5% level) post-hoc test was applied to physicochemical data to determine significant differences. The differences were considered statistically significant when *p* values were less than 0.05. These analyses were performed using Statistica 10 software (StatSoft, Tulsa, OK, USA).

## 3. Results and Discussion

### 3.1. Protocol for the Isolation of the White Wine Macromolecular Components

**Figure 1:**
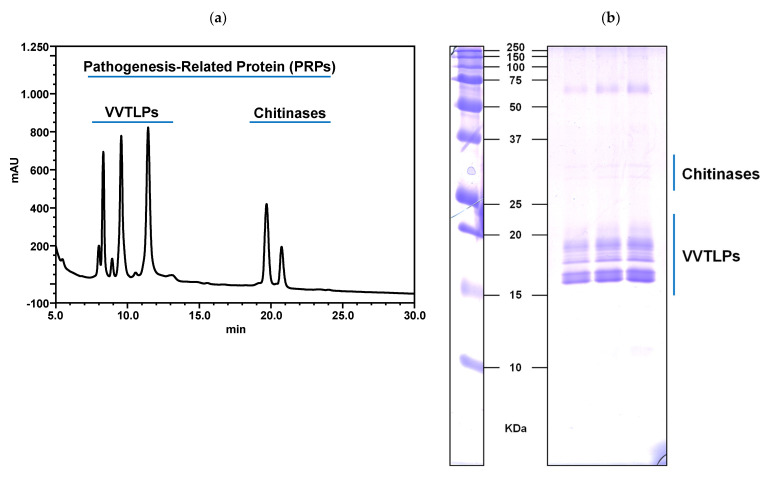
Reversed-phase high-performance liquid chromatography (HPLC) results for an example wine, showing the different pathogenesis-related proteins (PRPs), namely *Vitis vinifera* thaumatin-like (VVTL) and chitinases proteins. Absorbance was registered at 210 nm (**a**). Sodium dodecyl sulphate polyacrylamide gel electrophoresis (SDS-PAGE) of the same wine in increasing amount of protein (5, 7.5, and 10 µg), showing the above-mentioned proteins (**b**).

Proteins and polysaccharides are the two main and almost exclusive components of the macromolecular fraction of white wines. In white wines, they are present in low concentrations when compared to the low molecular components like tartaric acid, residual sugars, and even phenolic compounds. Typically, white wine protein contents range between 15 and 500 mg/L [9,14,29,49,62,63], and white wines polysaccharides from 50 to 150 mg/L [64,65]. The first step for studying the macromolecular components of wines is being able to concentrate and remove potential interfering compounds that would affect their characterisation, for example, in the analysis of proteins. The analysis of PRPs, the main culprits for protein instability in wines, have been widely performed by RP-HPLC. However, sample preparation for chromatographic separation varies between studies and according to their purpose. For quantitative purposes, direct injection has been used [50,66]. Other authors opted for prior protein precipitation using ammonium sulphate and subsequent hydrophobic interaction chromatography (HIC) fractionation before HPLC analysis [67]. Jaeckels et al. [48] used ultrafiltration to isolate and concentrate the high molecular weight material (HMWM) of wines (molecular mass cut-off of 10 kDa). In this work, our experimental design also included ultrafiltration for the isolation of the wine macromolecular components but with the addition of the chaotropic agent urea at 6 M concentration. This served to reduce the non-covalent interactions between high molecular weight components, particularly proteins, and phenolic compounds present in wine, thus eliminated in the ultrafiltration step and allowing the purification of the high molecular weight fraction for subsequent studies without interferences. This is confirmed by the sodium dodecyl sulphate polyacrylamide gel electrophoresis (SDS-PAGE) result (Figure 1) which shows well-resolved protein bands without the interference of phenolic compounds that could alter their electrophoretic profile [68]. Also, the analysis of the extracts by RP-HPLC did not allow us to observe the presence of the common white wine phenolic compounds, showing the efficiency in their removal. Therefore, this protocol was used in the isolation of the macromolecular (>10 kDa) components of white wines.

### 3.2. Effect of the Skin Contact on the Albariño White Wine Protein Content, Heat Stability, and Efficiency of Protein Stabilisation by Fungal Chitosan, k-Carrageenan, and Bentonite

**Figure 2:**
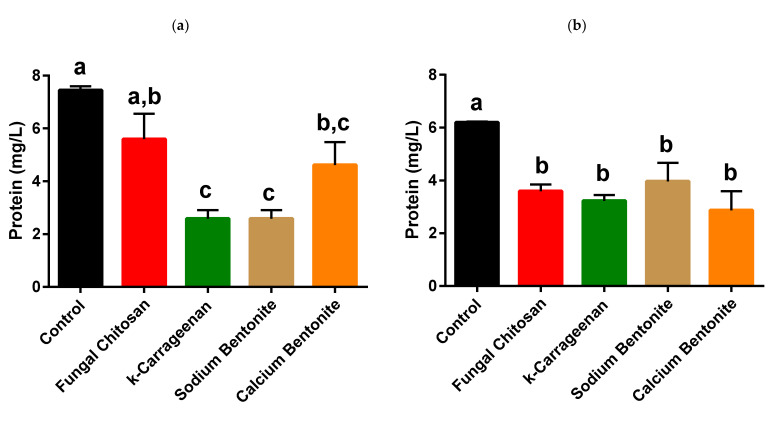
White wine protein content measured by the Bradford assay. (**a**) Albariño white wine without pre-fermentative skin maceration (−PFSM) and (**b**) Albariño white wine with pre-fermentative skin maceration (+PFSM). Fungal chitosan (100 g/hL), k-carrageenan (100 g/hL), sodium bentonite (120 g/hL), and calcium bentonite (120 g/hL). The columns values with the same letter are not statistically significant (Tukey *p* < 0.05).

**Figure 3:**
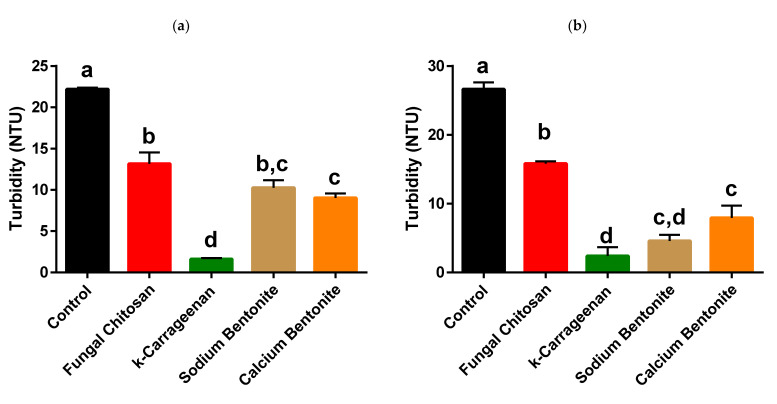
White wine protein stability measured by the increase in wine turbidity after heating for 30 min at 80 °C. (**a**) Albariño white wine without pre-fermentative skin maceration (−PFSM) and (**b**) Albariño white wine with pre-fermentative skin maceration (+PFSM) Fungal chitosan (100 g/hL), k-carrageenan (100 g/hL), sodium bentonite (120 g/hL), and calcium bentonite (120 g/hL). The columns values with the same letter are not statistically significant (Tukey *p* < 0.05).

The wine obtained without pre-fermentative skin maceration (−PFSM) presented a total protein content significantly higher than the wine obtained with pre-fermentative skin maceration (+PFSM; Figure 2). This can be due to the higher extraction of phenolic compounds from the skin during the pre-fermentative maceration which in turn can have insolubilised a higher amount of proteins when compared to the wines obtained without pre-fermentative maceration (further discussed below). Both wines presented significant protein instability, with the +PFSM wine showing a significantly higher protein instability, measured by the increase in wine turbidity after heating 30 min at 80 °C when compared to the −PFSM counterpart (Figure 3). To stabilise the wines concerning their protein instability, four fining agents were compared concerning their efficiencies: two bentonites, one sodium, and one calcium bentonite, and two polysaccharides, fungal chitosan, and k-carrageenan.

As can also be observed in Figure 3 the application of fungal chitosan (100 g/hL), k-carrageenan (100 g/hL), sodium bentonite (120 g/hL), and calcium bentonite (120 g/hL) decreased significantly the protein instability for both wines. Nevertheless, only the application of k-carrageenan was able to reduce the protein instability to stability levels (ΔNTU; Figure 3) [52,69]. The ability of k-carrageenan in increasing the heat stability of the wines was only observed when the wines were previously filtered to the determination of the turbidity. The centrifugation of wines before the heat stability test increased the wine turbidity (results not shown). This observation is in line with the results obtained by Ratnayake et al. [31], who observed that, after filtering, the k-carrageenan was able to further reduce the protein content of the wines. In both wines, sodium and calcium bentonite at the dosage used were not able to completely stabilise the wines, being more efficient in +PFSM than in −PFSM wine (Figure 3). The efficiency of both bentonites was not significantly different in stabilising the wines concerning protein stability. Of all the products tested, fungal chitosan was the least efficient product, presenting similar efficiencies in both wines (Figure 3). The efficiency of k-carrageenan for reducing protein instability in wines is in accordance with the results of Ratnayake et al. [31], who observed a similar performance for k-carrageenan and sodium bentonite. Nevertheless, in this work, k-carrageenan was more efficient than both sodium and calcium bentonites. The efficiency of chitosan was lower than that observed for sodium and calcium bentonites and much lower than that observed for k-carrageenan. This lower efficiency of chitosan is not in accordance with the results of Colangelo et al. [30], yet this difference may be related to the different protein stability tests used, as they used 60 °C temperature and in this work, we used 80 °C temperature for protein denaturation. Furthermore, it is known that Vitis vinifera thaumatin-like (VVTL) proteins (VVTLPs) are not denatured at 60 °C and are denatured at 80 °C [12,70].

To understand the differences observed in the protein stability of these two wines and the effect of the application of the different fining products, and especially the efficiency of k-carrageenan in the heat stabilisation of the wines, the total protein content was determined (Figure 2). The application of the different fining agents resulted in a significant decrease in the total protein content in +PFSM wine (Figure 2), whereas no significant results were found between the different treatments. On the other hand, for the −PFSM wine the application of fungal chitosan was not able to reduce significantly the total protein content (Figure 2). These results show that although the fining agents can decrease the total protein content of wines, depending on the wine matrix and fining agent, the decrease in total wine protein content does not allow us to explain the white wine protein stability (Figure 3). These results are in accordance with the results of Bayly and Berg [71] and Moretti and Berg [72] who found that the total protein content of wines was not related to their protein stability.

**Figure 4:**
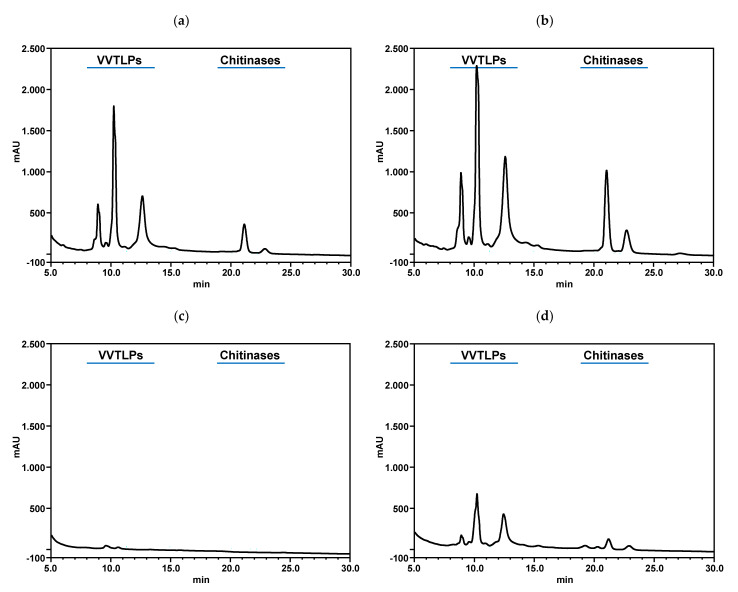

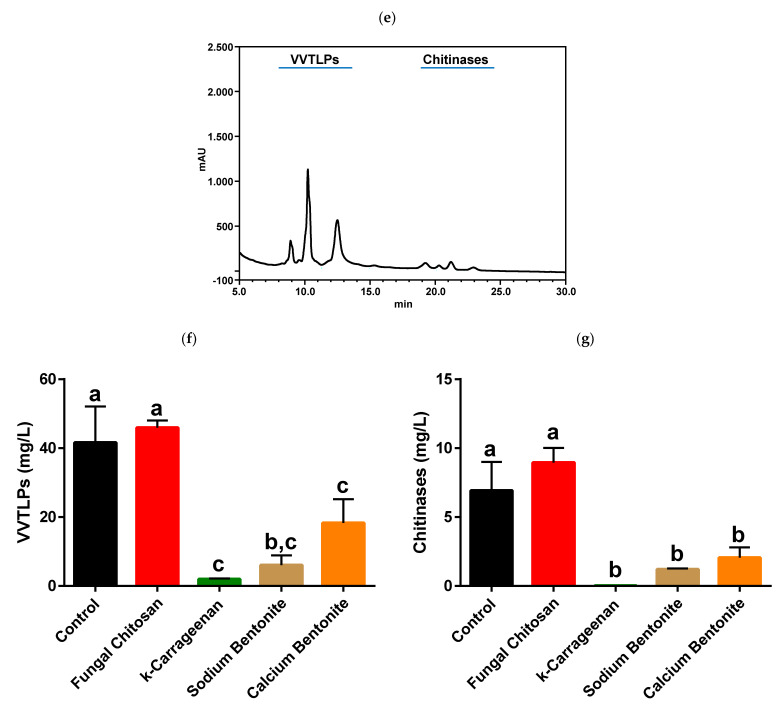
Reversed-phase HPLC results for the wine obtained without pre-fermentative skin maceration (−PFSM) and the impact of the different products applied for its protein stabilisation. (**a**) control wine without any additive; (**b**) after application of fungal chitosan at 100 g/hL; (**c**) after application of k-carrageenan at 100 g/hL; (**d**) after application of sodium bentonite at 120 g/hL; (**e**) after application of calcium bentonite at 120 g/hL. All chromatograms were obtained by analysis of a 5 mg/mL solution of the high molecular weight material (HMWM) after removal of the low molecular weight material by addition of 6 M urea and repeated ultrafiltration through a 10 kDa cut-off membrane, (**f**) *Vitis vinifera* thaumatin-like proteins (VVTLPs) concentration after application of fungal chitosan (100 g/hL), k-carrageenan (100 g/hL), sodium bentonite (120 g/hL), and calcium bentonite (120 g/hL, (**g**) chitinases concentration after application of fungal chitosan (100 g/hL), k-carrageenan (100 g/hL), sodium bentonite (120 g/hL), and calcium bentonite (120 g/hL). The columns values with the same letter are not statistically significant (Tukey *p* < 0.05).

**Figure 5:**
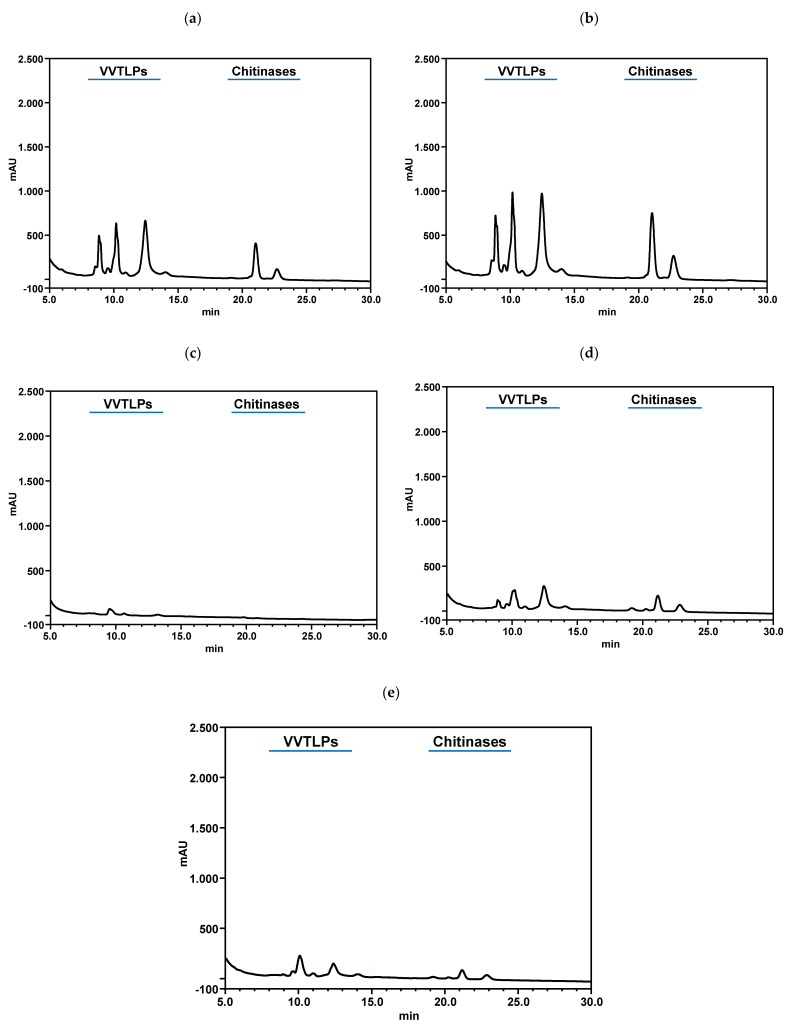

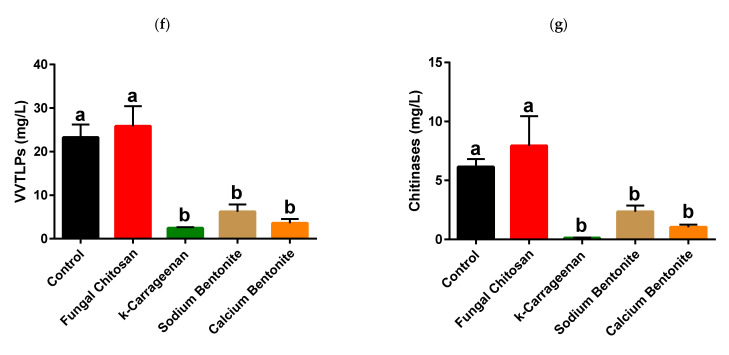
Reversed-phase HPLC results for the wine obtained with pre-fermentative skin maceration (+PFSM) and the impact of the different products applied for its protein stabilisation. (**a**) control wine without any additive; (**b**) after application of fungal chitosan at 100 g/hL; (**c**) after application of k-carrageenan at 100 g/hL; (**d**) after application of sodium bentonite at 120 g/hL; (**e**) after application of calcium bentonite at 120 g/hL. All chromatograms were obtained by analysis of a 5 mg/mL solution of the HMWM after removal of the low molecular weight material by addition of 6 M urea and repeated ultrafiltration through a 10 kDa cut-off membrane, (**f**) VVTLPs concentration after application of fungal chitosan (100 g/hL), k-carrageenan (100 g/hL), sodium bentonite (120 g/hL), and calcium bentonite (120 g/hL, (**g**) chitinases concentration after application of fungal chitosan (100 g/hL), k-carrageenan (100 g/hL), sodium bentonite (120 g/hL), and calcium bentonite (120 g/hL). The columns values with the same letter are not statistically significant (Tukey *p* < 0.05).

It has been shown that the most abundant haze-forming proteins include chitinases [73], along with VVTLPs and β-glucanases [74]. The wine protein instability may also be influenced by non-protein factors including the wine pH, ionic strength [75], ethanol content, concentrations of polysaccharides [26,68], polyphenols [76], and sulphates [17]. Therefore, the content of VVTLPs and chitinases of +PFSM and −PFSM wines was determined by RP-HPLC (Figure 4 and Figure 5). As can be observed, both wines contained significant amounts of VVTLPs and chitinases, with −PFSM wine presenting a significantly higher amount of both proteins, in line with the results obtained for the total wine protein content (Figure 2). The application of k-carrageenan, sodium, and calcium bentonite significantly decreased their contents when compared to control wine, with k-carrageenan showing the highest efficiency in the reduction of these two protein fractions for both wines (Figure 4 and Figure 5). The ability of sodium and calcium bentonite for reducing the content of VVTLPs and chitinases in wines has already been observed by different authors [12,21,68]. At wine pH, VVTLPs and chitinases present an overall positive charge, and bentonite is negatively charged, therefore the ability to remove VVTLPs and chitinases is attributed to the electrostatic interaction between these two proteins and bentonite [12]. The same mechanism can be hypothesised for the k-carrageenan. k-Carrageenan is composed of a galactan backbone of alternating 1,3-linked-β-D-galactopyranosyl and 3,6-anhydro-D-galactose residues with one SO_3_− residue per disaccharide; therefore, presenting a negative charge at wine pH. The ability of k-carrageenan in increasing the white wine protein stability is in accordance with the results of Cabello-Pasini et al. [39], Marangon et al. [15,40], and Ratnayake et al. [31]. Nevertheless, as mentioned before, k-carrageenan was only able to stabilise the wines after filtration, being observed an increase in wine turbidity when the wines are centrifuged. This observation agrees with Ratnayake et al. [31]. On the other hand, fungal chitosan was unable to reduce the contents of these two protein fractions when compared to control. This result is partially in accordance with Colangelo et al. [30]. These authors showed that chitosan was unable to remove VVTLPs, while able to reduce chitinases. This difference can be due to the chemical composition of the wines used in this study and to the relative amount of chitinases in the wines analysed. Therefore, the differences in the relative efficiency of chitosan, k-carrageenan, sodium bentonite, and calcium bentonite in the stabilisation of white wine protein precipitation can be explained by the relative removal efficiency of these two pathogenesis-related proteins, and the different behaviour observed in the two wines obtained by different treatments is also explained by the relative amount of these proteins in both wines.

### 3.3. Effect of k-Carrageenan Addition on Wine Filterability

**Figure 6:**
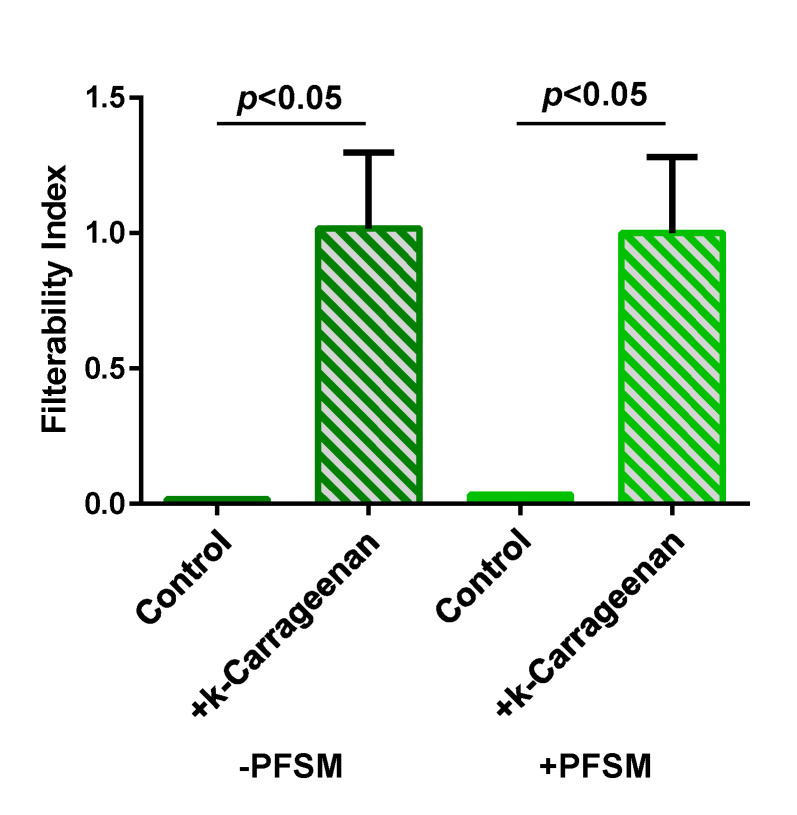
Effect of k-carrageenan addition to the Albariño white wines obtained without pre-fermentative skin maceration (−PFSM) and with pre-fermentative skin maceration (+PFSM).

As described above, the wine protein stabilisation using k-carrageenan was only possible after wine filtration, and therefore the impact of its application on the wine filterability index was determined (Figure 6). The addition of k-carrageenan to both wines significantly increased their filterability index. These results are in accordance with Marangon et al. [15] and Ratnayake et al. [31] indicating that the addition of k-carrageenan to wines decreases its filterability. Nevertheless, the filterability index obtained after the addition of k-carrageenan was well below 20, a value that is taken as a limit for the wine filterability. In this sense, no problems of filterability are anticipated with the application of k-carrageenan to wines.

### 3.4. Effect of the Skin Contact on the Albariño White Wine Phenolic Composition and Chromatic Characteristics, and Effect of the Addition of Fungal Chitosan, k-Carrageenan and Bentonite

**Figure 7:**
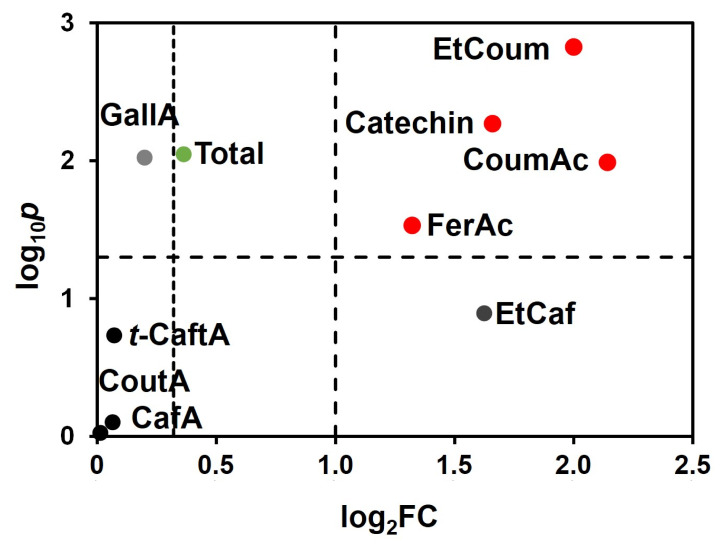
Volcano plot representing the statistical significance (p-values) on the Student t-test and fold change (FC) for the phenolic content of Albariño white wine produced without pre-fermentative skin maceration (−PFSM) versus wine produced with pre-fermentative skin maceration (+PFSM). GallA—Gallic acid, t-CaftA—trans-Caftaric acid, CoutA—Coutaric acid, CoumAc—p-Coumaric acid, FerAc—Ferulic acid, CafA—Caffeic acid, EtCoum—Ethyl ester of p-coumarate, EtCaf—Ethyl ester of caffeate, Total—Total phenolic composition.

In order to study the effect of the white wine obtained without pre-fermentative skin maceration (−PFSM) and with pre-fermentative skin maceration (+PFSM) in the phenolic composition of the wines and its possible relation to the total protein content, the phenolic composition of wines was determined using a colourimetric method and the individual phenolic compounds were analysed by RP-HPLC [58,59,60]. The chromatic characteristics were determined according to the CIELa*b* method [56]. As can be observed in Table 2, +PFSM wine presented a significantly higher amount of total phenols, flavonoid phenols, and non-flavonoid phenols when compared to the −PFSM wine. In line with the highest extraction of phenolic compounds observed for the +PFSM wine, this wine presented a significantly lower lightness (L*) and higher yellow colour (b* value) when compared with the −PFSM wine (Table 2). Table 3 presents the individual phenolic compounds for both wines and in Figure 7 is presented the volcano plot showing the representation of the log of the probability obtained by the t-test for each phenolic compound in function of the fold change in the individual and total phenolics concentration determined by HPLC. As can be observed in Table 3 and Figure 7, the +PFSM wine showed a significant increase in the total phenolics extracted (an increase of 29%). The main phenolic compounds whose concentration increased by at least double were p-coumaric acid, catechin, the ethyl ester of p-coumaric acid, and ferulic acid. In this work, we only determined the monomeric flavonols, showing a significant increase in the catechin levels by maceration, probably resulting from the increased extraction from skins and seeds [77]. It is expected that an increase in condensed tannins will also occur; nevertheless, their levels were not determined by this method. Therefore, the lower amount of protein present in the final +PFSM wine is probably related to the higher levels of phenolic compounds in this wine.

Table 3 also shows the phenolic composition of the two white wines after application of the fungal chitosan, k-carrageenan, sodium, and calcium bentonite. As can be observed, the application of the different fining agents resulted in a significant but small decrease in the total phenols of the wines, the decrease being generally smaller for the +PFSM wine (from 11% for the fungal chitosan to 8% for the calcium bentonite and k-carrageenan) than −PFSM (from 23% for fungal chitosan and calcium bentonite to 10% for k-carrageenan). Again, depending on the wine matrix, the effect of the application of the different fining agents was different; nevertheless, for the application of fungal chitosan, a significant decrease in the b* values and an increase in the L* values were observed. The application of sodium and calcium bentonites did not affect the L* and b* values for the +PFSM wine, although for the −PFSM wine a significant decrease in the L* and a* values was observed. In general, the impact of these fining agents on the chromatic characteristics of both wines was small.

### 3.5. Effect of the Skin Contact on the Albariño Wine Polysaccharides Composition and Effect of the Addition of Fungal Chitosan, k-Carrageenan and Bentonite

Table 4 shows the total polysaccharides content of both wines and their sugar composition. As can be observed the white wine obtained with pre-fermentative skin maceration increased significantly the total polysaccharide content of the wines (28% increase). Both wines contained significant amounts of mannose, which was the main sugar with a relative abundance of 28 to 43% by weight of the total sugars present in +PFSM and −PFSM wines, respectively. This is probably derived from the mannoproteins released into the wines by Saccharomyces yeast during fermentation [77]. Nonetheless, the amount of mannose present in both wines was not significantly different (Table 4). Galacturonic acid was the second most abundant sugar accounting for nearly 22% of the total polysaccharides of both wines. Its amount was significantly higher for +PFSM wine (28% increase; Table 4). Galacturonic acid and rhamnose are probably derived from the rhamnogalacturonan I and rhamnogalacturonan II pectic polysaccharides originating from grapes [78,79]. As observed for galacturonic acid residues, the level of rhamnose residues present in wine also increased significantly with the pre-fermentative skin maceration winemaking technology (103% increase; Table 4), with the rhamnose residues accounting for 5 and 8% of the total polysaccharides extracted for +PFSM and −PFSM wines, respectively. Galactose residues account for a significant amount of the polysaccharides extracted with both winemaking techniques, accounting for nearly 14% of the total polysaccharides. Galactose residues along with arabinose residues are derived from the type I and type II arabinogalactans from grapes [41]. The arabinose residues extracted increased significantly for the wines obtained with the pre-fermentative skin maceration (115% increase) representing 4% to 7% of the wine polysaccharides obtained without and with pre-fermentative skin maceration, respectively. The most significant changes observed in the wine polysaccharide composition were related to the significant increase in the glucuronic acid residues (181% increase) and the appearance of a significant amount of xylose residues in the +PFSM wine; xylose was not detected in the −PFSM wine. Glucuronic acid residues are known to be constituents of grape-derived wine type II arabinogalactans where they can be present in the arabinose side chains of arabinogalactans. This increase can be due to the extraction of more glucuronic acid-containing type II arabinogalactans [79] when the wines were obtained with pre-fermentative skin maceration. Xylose residues are probably derived from grape skin xylans [80,81]. Therefore, these results show that the pre-fermentative skin maceration process can increase the total polysaccharides present in wine derived from the grape skins.

The addition of chitosan drastically decreased the polysaccharide content of the wines: 59% in the −PFSM wine and 65% in +PFSM wine (Table 4). Although chitosan application has reduced the content of all polysaccharides, the sugars with a higher reduction were galacturonic acid and rhamnose, as well as glucuronic acid, showing that the biggest impact of chitosan was on the polysaccharides bearing potential negative charge due to the presence of these two uronic acids. Therefore, we hypothesised that their removal is due to the electrostatic interaction between the positively charged chitosan and the negatively charged polysaccharides, probably rhamnogalacturonan I and II and type II arabinogalactans as a decrease in the galactose and arabinose residues was also observed. This trend is observed for both wines, with and without pre-fermentative skin maceration. Yeast mannoproteins, grape arabinogalactan-proteins (AGP), and rhamnogalacturonan RG-II—carried negative charges in the wine pH range. The net charge density of yeast mannoproteins was shown to be related to their phosphorus content and absolute charge densities of AGP and RG-II were related to the dissociation of the carboxylic functions of their uronic acids [82]. Sodium bentonite was the second treatment in reducing the levels of polysaccharides from both wines. In the −PFSM wine, sodium bentonite resulted in a decrease of 59% in the total polysaccharides, and for the +PFSM wine, the reduction was 27% (Table 4). For the −PFSM wine, the polysaccharide removal profile was similar to that observed for the fungal chitosan, but for the +PFSM wine, the main affected sugar residue was mannose, allowing us to infer that it is mainly removing wine mannoproteins. The impact of the application of calcium bentonite was lower than that observed for the application of sodium bentonite, nevertheless, its use also significantly reduced the total levels of polysaccharides in the final wines, being observed a reduction of 19% and 16% for the −PFSM and +PFSM wines (Table 4). k-Carrageenan application increased on average, although not significantly, the levels of polysaccharides present in the wine, being mainly observed a significant increase in the levels of galactose and glucose residues, showing that after application and filtration some polysaccharides from the k-carrageenan preparation used remained in the wine. As can be observed in Table 4, and in contrast to the other fining agents used, k-carrageenan was the only fining agent that did not affect significantly the levels of the remaining polysaccharides (Table 4). Of all the fining agents used, k-carrageenan showed a higher specificity in the removal of the heat unstable proteins without impacting polysaccharide composition.

## 4. Conclusions

Pre-fermentative skin maceration increased the levels of phenolic compounds and polysaccharides extracted and reduced the amount of protein extracted, especially of the pathogenesis-related proteins, namely the *Vitis vinifera* thaumatin-like proteins and chitinases. Although the total protein and PRPs of the Albariño wine obtained by pre-fermentative skin maceration were lower, it showed a significantly higher protein instability. When the efficiency of sodium and calcium bentonite, fungal chitosan, and k-carrageenan for wine protein stabilisation were compared it was observed that k-carrageenan reduced the wine protein instability and the content of *Vitis vinifera* thaumatin-like proteins and chitinases and was effective for both wines. Sodium and calcium bentonites were also able to increase the wine protein stability but only for the wine obtained without pre-fermentative skin maceration. They were less efficient in decreasing the levels of *Vitis vinifera* thaumatin-like proteins and chitinases in both white wines when compared to k-carrageenan. Fungal chitosan was unable to heat stabilise the wines and did not change the levels of *Vitis vinifera* thaumatin-like proteins and chitinases. On the other hand, fungal chitosan decreased by ~60% the levels of wine polysaccharides, the same being observed for sodium and calcium bentonite, although these two fining agents being less deleterious. k-Carrageenan did not decrease significantly the levels of polysaccharides present in both wines. The use of k-carrageenan decreased slightly the wine filterability, but at a level that does not affect its filterability in practical terms. Therefore, the use of k-carrageenan for white wine protein stabilisation is a good approach as its impact on the macromolecular components is more specific, i.e., it significantly reduced the levels of the PRPs of wines without impacting the polysaccharide composition, with similar or even better efficiency than sodium bentonite. The use of skin maceration in the production of Albariño white wines showed a positive influence on the levels of polysaccharides and phenolic compounds and this can have a positive influence on the sensory characteristics of the final wines. Nevertheless, future work using sensory analysis is needed to access the impact of these changes on the quality of the final wines.

## Figures and Tables

**Table 1:** Physicochemical characteristics from the Albariño monovarietal white wine with and without pre-fermentative skin maceration (PFSM) used in the experiments.

Albariño Monovarietal White Wine	With Pre-Fermentative Skin Maceration (+PFSM)	Without Pre-Fermentative Skin Maceration (−PFSM)
Alcohol content (% *v*/*v*)	13.6	14.3
Density at 20 °C (g/cm^3^)	0.9888	0.9883
Titratable acidity (expressed as g/L tartaric acid)	6.2	7.3
pH	3.81	3.52
Volatile acidity (expressed as g/L acetic acid)	0.42	0.45
Protein instability (Heat-test, ΔNTU)	26.6	22.2

**Table 2:** Total phenols (mg/L), flavonoid phenols (mg/L), non-flavonoid phenols (mg/L), white wine colour (Abs420 nm), and chromatic characteristics of Albariño white wines obtained without and with pre-fermentative skin maceration, and effect of the application of fungal chitosan (100 g/hL), k-carrageenan (100 g/hL), sodium (120 g/hL) and calcium bentonites (120 g/hL).

	Total Phenols	Flavonoid Phenols	Non-Flavonoid Phenols	Abs _420 nm_	L*	a*	b*	ΔE*
**Without pre-fermentative skin contact**								
Control	39 ± 1	22 ± 2 ^a^	17 ± 1 ^a^	0.079 ± 0.006 ^a^	98.2 ± 0.5 ^a^	0.14 ± 0.08 ^a^	4.89 ± 0.08	
Fungal Chitosan	35 ± 0	21 ± 0 ^a,c^	15 ± 1 ^a^	0.076 ± 0.014 ^a,b^	99.1 ± 0.4 ^a,b^	0.12 ± 0.08 ^a^	3.84 ± 0.96	1.43 ± 1.13
k-Carrageenan	38 ± 1	22 ± 0 ^a^	16 ± 0 ^a^	0.079 ± 0.002 ^a^	98.1 ± 0.2 ^a^	0.16 ± 0.07 ^a^	4.78 ± 0.03	0.13 ± 0.07
Sodium Bentonite	38 ± 0	17 ± 1 ^b,c^	21 ± 0 ^b^	0.075 ± 0.002 ^a,b^	99.3 ± 0.4 ^a,b^	−0.40 ± 0.04 ^b^	4.00 ± 0.28	1.62 ± 0.84
Calcium Bentonite	39 ± 2	24 ± 1 ^a^	15 ± 1 ^a^	0.048 ± 0.003 ^b^	99.9 ± 0.1 ^b^	−0.46 ± 0.02 ^b^	3.79 ± 0.08	1.07 ± 0.30
**ANOVA**	0.0752	0.0157	0.0037	0.0279	0.0152	0.0003	0.1450	0.3059
**With pre-fermentative skin contact**								
Control	59 ± 0 ^a^	32 ± 0 ^a^	28 ± 0 ^a^	0.117 ± 0.008 ^a^	97.8 ± 0.5	0.77 ± 0.02	6.78 ± 0.17 ^a^	
Fungal Chitosan	52 ± 0 ^b,c^	33 ± 1 ^a^	19 ± 1 ^b^	0.084 ± 0.008 ^b^	98.1 ± 0.2	0.70 ± 0.12	5.32 ± 0.49 ^b^	1.52 ± 0.31
k-Carrageenan	54 ± 0 ^b^	33 ± 1 ^a^	21 ± 0 ^b,c^	0.116 ± 0.002 ^a^	97.6 ± 0.1	0.81 ± 0.04	6.68 ± 0.11 ^a^	0.19 ± 0.11
Sodium Bentonite	48 ± 0 ^c^	26 ± 1 ^b,c^	22 ± 0 ^c^	0.107 ± 0.010 ^a,b^	97.3 ± 0.7	1.03 ± 0.14	6.49 ± 0.15 ^a^	0.74 ± 0.48
Calcium Bentonite	52 ± 0 ^b,c^	29 ± 1 ^a,c^	22 ± 0 ^c^	0.102 ± 0.005 ^a,b^	97.6 ± 0.2	0.99 ± 0.07	6.42 ± 0.23 ^a^	0.59 ± 0.27
**ANOVA**	0.0001	0.0023	0.0002	0.0280	0.4670	0.0534	0.0143	0.0575
***t*** **-test**	0.0016	0.0206	0.0051	0.0329	0.508	0.0085	0.0049	

Values are presented as mean ± standard deviation; Means within a comun for each wine followed by the same letter are not significantly different (Tukey *p* < 0.05). L* for the lightness from black (0) to white (100), a* from green (−) to red (+), and b* from blue (−) to yellow (+), ΔE*—Color difference in relation to control wine. Analysis of variance (ANOVA). *t*-test—Student’s *t*-test.

**Table 3:** Phenolic composition (mg/L) determined by reversed phase-high performance liquid chromatography (RP-HPLC) of Albariño white wine wines obtained without and with pre-fermentative skin maceration and effect of the application of fungal chitosan (100 g/hL), k-carrageenan (100 g/hL), sodium (120 g/hL) and calcium bentonites (120 g/hL).

	Gallic Acid	Catechin	*t*-Caftaric Acid	Coutaric Acid	Caffeic Acid	*p*-Coumaric Acid	Ferulic Acid	Ethyl Ester of Caffeate	Ethyl Ester of *p*-Coumarate	Total
**Without pre-fermentative skin maceration**										
Control	50.0 ± 0.9	7.06 ± 1.48	23.8 ± 0.3	21.7 ± 1.6	6.48 ± 0.74	1.84 ± 0.80	0.06 ± 0.01	0.12 ± 0.14	0.12 ± 0.00	111.4 ± 3.5 ^a^
Fungal Chitosan	46.0 ± 0.1	6.78 ± 0.34	23.6 ± 0.6	15.5 ± 1.8	5.89 ± 0.09	1.07 ± 0.23	0.04 ± 0.02	0.03 ± 0.00	0.10 ± 0.02	99.1 ± 1.1 ^b^
k-Carrageenan	48.3 ± 1.6	6.06 ± 0.06	23.3 ± 0.3	16.2 ± 2.8	6.14 ± 0.13	1.66 ± 0.22	0.05 ± 0.01	0.06 ± 0.04	0.08 ± 0.06	102.0 ± 0.7 ^b^
Sodium Bentonite	49.7 ± 0.2	5.58 ± 0.61	23.7 ± 1.1	14.9 ± 0.1	6.53 ± 0.05	1.33 ± 0.04	0.04 ± 0.01	0.03 ± 0.00	0.05 ± 0.04	101.9 ± 0.6 ^b^
Calcium Bentonite	52.7 ± 6.9	3.70 ± 0.99	22..9 ± 0.5	15.6 ± 0.4	6.14 ± 0.41	1.19 ± 0.23	0.03 ± 0.01	0.06 ± 0.06	0.06 ± 0.01	102.5 ± 2.6 ^b^
**ANOVA**	0.4201	0.0573	0.6431	0.0428	0.5037	0.3793	0.3008	0.7122	0.3445	0.0122
**With pre-fermentative skin maceration**										
Control	57.4 ± 0.5 ^a^	22.3 ± 0.6	25.0 ± 0.8	22.7 ± 4.4	6.54 ± 0.80	8.12 ± 0.43	0.15 ± 0.02	0.37 ± 0.01	0.48 ± 0.02	143.3 ± 2.5 ^a^
Fungal Chitosan	31.5 ± 4.9 ^b^	22.4 ± 0.2	26.0 ± 3.5	20.4 ± 5.6	6.11 ± 0.44	5.29 ± 1.03	0.06 ± 0.01	0.32 ± 0.01	0.44 ± 0.07	112.9 ± 1.3 ^c^
k-Carrageenan	50.5 ± 4.6 ^a^	22.7 ± 1.9	25.9 ± 3.2	19.7 ± 0.2	5.86 ± 0.15	5.48 ± 3.58	0.07 ± 0.06	0.36 ± 0.07	0.47 ± 0.06	129.6 ± 3.4 ^b,c^
Sodium Bentonite	47.1 ± 2.1 ^a^	20.7 ± 0.2	25.1 ± 0.5	18.5 ± 2.1	5.83 ± 0.08	6.88 ± 0.43	0.13 ± 0.08	0.36 ± 0.02	0.13 ± 0.12	124.7 ± 0.4 ^b^
Calcium Bentonite	35.3 ± 0.9 ^b^	20.4 ± 0.6	24.4 ± 0.6	19.6 ± 1.1	5.70 ± 0.03	4.93 ± 0.89	0.13 ± 0.07	0.29 ± 0.09	0.40 ± 0.31	118.4 ± 5.3 ^b^
**ANOVA**	0.0021	0.1639	0.1883	0.7753	0.3888	0.4206	0.4655	0.5520	0.2718	0.0012
***t*** **-test**	0.0095	0.0054	0.1854	0.7911	0.9450	0.0103	0.0295	0.1280	0.0015	0.0090

Values are presented as mean ± standard deviation; Means within a comun for each wine followed by the same letter are not significantly different (Tukey *p* < 0.05). Analysis of variance (ANOVA). *t*-test—Student’s *t*-test.

**Table 4:** Sugar composition (mg/L) of polysaccharides from Albariño white wines produced without and with pre-fermentative skin maceration recovered by ultrafiltration and effect of the application of fungal chitosan (100 g/hL), k-carrageenan (100 g/hL), sodium (120 g/hL) and calcium bentonites (120 g/hL).

	Fuc	Rha	Ara	GlcN	Gal	Glc	Xyl	Man	GalA	GlcA	Total
**Without pre-fermentative skin contact**											
Control	0.25 ± 0.03 ^a^	2.79 ± 0.15 ^a^	2.46 ± 0.10 ^a^	0.91 ± 0.17 ^a^	9.17 ± 0.55 ^a^	2.04 ± 0.06 ^a^	n.d.	27.8 ± 0.1 ^a^	12.4 ± 0.2 ^a^	3.73 ± 0.33 ^a^	61.6 ± 1.0 ^a^
Fungal Chitosan	n.d. ^c^	0.59 ± 0.13 ^c^	1.00 ± 0.06 ^b^	0.66 ± 0.17 ^a,b^	5.25 ± 0.80 ^b^	1.87 ± 0.44 ^a,c^	n.d.	13.4 ± 1.6 ^b^	1.28 ± 0.53 ^b^	0.90 ± 0.01 ^b^	29.0 ± 1.0 ^c^
k-Carrageenan	0.25 ± 0.02 ^a^	2.79 ± 0.15 ^a^	2.72 ± 0.33 ^a^	1.06 ± 0.09 ^a^	13.3 ± 1.2 ^c^	3.31 ± 0.39 ^b^	n.d.	24.8 ± 1.9 ^a^	10.4 ± 2.3 ^a^	3.93 ± 0.12 ^a^	65.6 ± 2.2 ^a^
Sodium Bentonite	0.11 ± 0.01 ^b^	1.33 ± 0.11 ^b^	1.38 ± 0.21 ^b^	0.35 ± 0.05 ^b^	5.40 ± 0.85 ^b^	0.89 ± 0.21 ^c^	n.d.	11.9 ± 1.5 ^b^	2.18 ± 0.91 ^b^	1.86 ± 0.06 ^c^	25.4 ± 3.8 ^c^
Calcium Bentonite	0.17 ± 0.04 ^a,b^	2.36 ± 0.18 ^a^	2.64 ± 0.19 ^a^	0.70 ± 0.06 ^a,b^	10.4 ± 0.3 ^a,c^	1.52 ± 0.13 ^a,c^	n.d.	23.5 ± 1.3 ^a^	4.83 ± 0.11 ^b^	3.50 ± 0.25 ^a^	49.6 ± 2.0 ^b^
**ANOVA**	0.0007	<0.0001	0.0010	0.0129	0.0007	0.0031	-	0.0003	0.0006	<0.0001	<0.0001
**With pre-fermentative skin contact**											
Control	0.36 ± 0.02 ^a^	5.68 ± 0.01 ^a^	5.30 ± 0.15 ^b^	0.87 ± 0.06 ^a^	11.7 ± 0.5 ^a^	2.62 ± 0.55 ^a^	2.75 ± 0.07 ^a^	23.2 ± 1.4 ^a^	15.9 ± 1.0 ^a^	10.5 ± 1.5 ^a^	78.8 ± 2.2 ^a^
Fungal Chitosan	0.02 ± 0.01 ^c^	1.50 ± 0.02 ^b^	2.25 ± 0.24 ^c^	0.44 ± 0.07 ^b^	5.57 ± 0.84 ^b^	1.97 ± 0.15 ^a^	0.97 ± 0.16 ^b^	9.79 ± 1.91 ^b^	3.02 ± 1.07 ^b^	1.86 ± 0.22 ^b^	27.4 ± 2.6 ^b^
k-Carrageenan	0.38 ± 0.02 ^a^	5.08 ± 0.25 ^a^	6.34 ± 0.31 ^a^	0.84 ± 0.02 ^a^	19.4 ± 0.3 ^c^	4.93 ± 1.35 ^b^	3.92 ± 0.48 ^c^	28.4 ± 1.0 ^c^	16.0 ± 3.0 ^a^	10.2 ± 1.5 ^a^	95.8 ± 1.3 ^c^
Sodium Bentonite	0.27 ± 0.02 ^b^	4.39 ± 0.29 ^a^	4.44 ± 0.06 ^b^	0.46 ± 0.02 ^b^	10.8 ± 0.5 ^a^	1.55 ± 0.05 ^a^	1.57 ± 0.08 ^b^	15.3 ± 0.4 ^d^	12.3 ± 0.2 ^a^	6.56 ± 0.42 ^a,c^	57.6 ± 0.9 ^d^
Calcium Bentonite	0.30 ± 0.03 ^a,b^	5.92 ± 0.96 ^a^	5.23 ± 0.30 ^b^	0.47 ± 0.08 ^b^	13.5 ± 0.04 ^a^	1.86 ± 0.31 ^a^	2.20 ± 0.05 ^a^	18.9 ± 0.1 ^a,d^	11.7 ± 1.1 ^a^	5.88 ± 0.70 ^b,c^	65.9 ± 1.9 ^e^
**ANOVA**	<0.0001	0.0011	<0.0001	0.0010	<0.0001	0.0073	0.0004	<0.0001	0.0021	0.0018	<0.0001
***t*** **-test**	0.0497	0.0014	0.0020	0.7834	0.0406	0.2764	0.0001	0.0435	0.0399	0.0248	0.0097

Values are presented as mean ± standard deviation; Means within a comun for each wine followed by the same letter are not significantly different (Tukey *p* < 0.05). Analysis of variance (ANOVA). *t*-test—Student’s *t*-test. Fuc—fucose, Rha—rhamnose, Ara—arabinose, GlcN—glucosamine, Gal—galactose, Glu—glucose, Xyl—xylose, Man—mannose; GalA—galacturonic acid; GlcA—glucuronic acid.
